# CRISPR/Cas-mediated knock-in via non-homologous end-joining in the crustacean *Daphnia magna*

**DOI:** 10.1371/journal.pone.0186112

**Published:** 2017-10-18

**Authors:** Hitoshi Kumagai, Takashi Nakanishi, Tomoaki Matsuura, Yasuhiko Kato, Hajime Watanabe

**Affiliations:** 1 Department of Biotechnology, Graduate School of Engineering, Osaka University, 2–1 Yamadaoka, Suita, Osaka, Japan; 2 Frontier Research Base for Global Young Researchers, Graduate School of Engineering, Osaka University, 2–1 Yamadaoka, Suita, Osaka, Japan; Guangzhou Institutes of Biomedicine and Health, CHINA

## Abstract

The clustered regularly interspaced short palindromic repeats (CRISPR)/CRISPR-associated system (Cas) is widely used for mediating the knock-in of foreign DNA into the genomes of various organisms. Here, we report a process of CRISPR/Cas-mediated knock-in via non-homologous end joining by the direct injection of Cas9/gRNA ribonucleoproteins (RNPs) in the crustacean *Daphnia magna*, which is a model organism for studies on toxicology, ecology, and evolution. First, we confirmed the cleavage activity of Cas9 RNPs comprising purified Cas9 proteins and gRNAs in *D*. *magna*. We used a gRNA that targets exon 10 of the *eyeless* gene. Cas9 proteins were incubated with the gRNAs and the resulting Cas9 RNPs were injected into *D*. *magna* eggs, which led to a typical phenotype of the *eyeless* mutant, i.e., eye deformity. The somatic and heritable mutagenesis efficiencies were up to 96% and 40%, respectively. Second, we tested the CRISPR/Cas-mediated knock-in of a plasmid by the injection of Cas9 RNPs. The donor DNA plasmid harboring the fluorescent reporter gene was designed to contain the gRNA recognition site. The co-injection of Cas9 RNPs together with the donor DNAs resulted in generation of one founder animal that produced fluorescent progenies. This transgenic *Daphnia* had donor DNA at the targeted genomic site, which suggested the concurrent cleavage of the injected plasmid DNA and genomic DNA. Owing to its simplicity and ease of experimental design, we suggest that the CRISPR/Cas-mediated knock-in method represents a promising tool for studying functional genomics in *D*. *magna*.

## Introduction

*Daphnia*, a genus of water fleas, are small planktonic crustaceans found in freshwater habitats. They have long been used as a model for studies on evolution and ecology [1,2], since they occupy an important position in the aquatic food chain and show a high degree of phenotypic plasticity. *Daphnia* also serve as a model for understanding reproductive strategies because they produce parthenogenetic eggs under favorable environmental conditions, but produce sexual eggs, called resting eggs, under adverse environmental conditions [3–5]. Moreover, *Daphnia* are suitable for laboratory experimentation because they are easy to culture and observe, given their high fecundity and transparent body. Sequence databases including expressed sequence tags and whole genomes are available for two *Daphnia* species (*D*. *magna* [6,7] and *D*. *pulex* [8]), and their transcriptomes have also been sequenced [9,10]. These recent advances in *Daphnia* genomics have additionally rendered them useful as model organisms for studies on ecological and toxicological genomics. Several methods for the functional analysis of genes, such as RNA interference [11], overexpression [12], and plasmid integration [13], have been developed for *Daphnia*.

Artificial nucleases can induce site-specific double strand breaks (DSBs) in a genome, aiding the knock-out and knock-in of target genes [14,15]. They can be classified into two groups: i) Custom-designed artificial nucleases and ii) RNA-guided nucleases. The custom-designed nucleases such as zinc finger nucleases (ZFNs) and transcription activator-like effector nucleases (TALENs) consist of a customizable DNA-binding domain and a FokI nuclease domain [16]. The dimerization of these domains is required for creating DSBs at the target site [17]. RNA-guided nucleases are based on a bacterial immune system called the clustered regularly interspaced short palindromic repeats (CRISPR)/CRISPR-associated system (Cas), which needs two components, namely custom single guide RNAs and Cas9 nucleases [18]. The single guide RNAs direct the Cas9 nucleases to the target site to create DSBs. After the cleavage of DNA, the break is repaired by either non-homologous end joining (NHEJ) or homologous recombination (HR). In knock-out experiments, NHEJ repair incorporates random insertions or deletions (indels) at the target site. In knock-in experiments, NHEJ allows a long exogenous DNA fragment to be integrated into the cleaved site, although the junction between the integrated DNA and the host genome often contains indels [19–21]. HR allows the precise integration of foreign genes into the host genome; however, in contrast to NHEJ-mediated knock-in, the maximum possible length of the integrated sequence is limited. The experimental design of CRISPR/Cas is simpler and rapid than that of ZFNs or TALENs [15]. Therefore, CRISPR/Cas is more widely used for genome editing both in model and non-model organisms [22–24].

We previously established a CRISPR/Cas-mediated knock-out method in *D*. *magna* [25]. We utilized the *eyeless* (*ey*) gene as a marker gene for targeted mutagenesis because the impairment of the *D*. *magna eyeless* (*Dma-ey*) gene results in a clearly discernible deformity of the compound eye [25]. To target the homeobox region, we used two gRNAs, which were designed to bind the sense strand from exon 8 and the anti-sense strand from exon 10, respectively [25]. We co-injected the two gRNAs together with an *in vitro*-synthesized *Cas9* mRNA into parthenogenetic female eggs. This approach introduced indels in the target gene, thereby deforming the compound eye. However, this method had two disadvantages: (1) *Cas9* mRNA preparation is laborious and time-consuming, and (2) the heritable mutagenesis efficiency of 8% was 2.5 times lower compared to the TALEN-based method, which cleaves exon 10 of *Dma-ey* [26]. Thus, for knock-in experiments, we used the TALEN-based method to introduce foreign DNA into the *Dma-ey* locus via HR and NHEJ [27,28].

Recently, several groups have reported that the direct injection of Cas9 ribonucleoproteins (RNPs) comprising purified Cas9 proteins and gRNAs, rather than the co-injection of *Cas9* mRNAs and gRNAs, increases the knock-out efficiency in various hosts including nematodes, mice, and mammalian cells [29–31]. In addition, Cas9 RNPs are more efficient in integrating exogenous DNA into the genomes of human cells and nematodes [32,33]. Therefore, we hypothesized that Cas9 RNP injection may allow us to knock-in foreign DNA sequences into the *Daphnia* genome. To test this hypothesis, we first confirmed the cleavage activity of the *Dma-ey*-targeting Cas9 RNPs. Second, using these Cas9 RNPs, we successfully inserted plasmid DNA into the *Dma-ey* locus. Owing to its high efficiency, simplicity, and ease of design, the CRISPR/Cas mediated knock-in method can accelerate gene function analysis in *D*. *magna*.

## Results

### Knock-out of the *Dma-ey* gene by the injection of Cas9 RNPs

**Fig 1:**
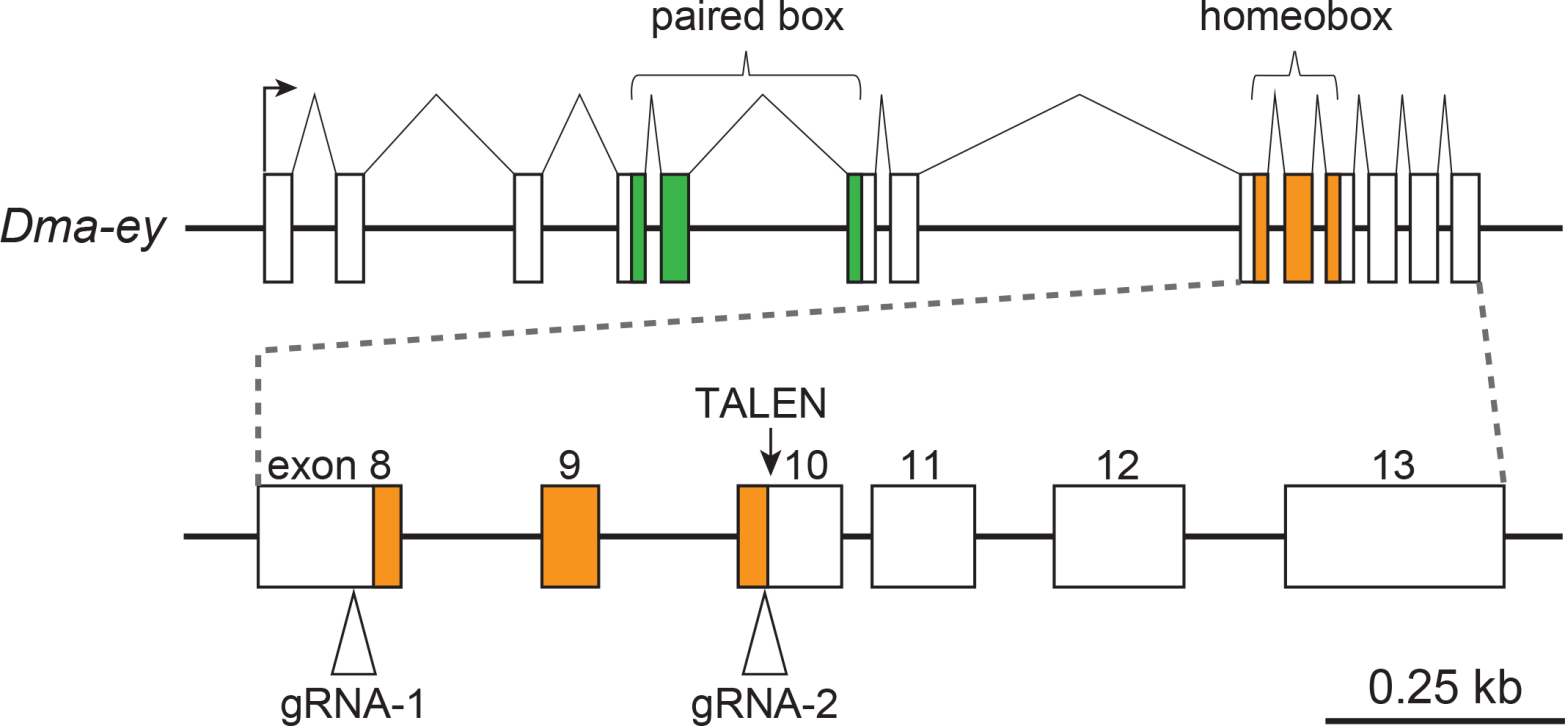
Design of gRNAs for the knock-out at the *Dma-ey* locus. This figure was drawn based on a previously published schematic illustration [25]. The arrowheads indicate the recognition sites of gRNA-1 and gRNA-2. An arrow indicates the target site of TALEN as previously reported [26].

We examined the target cleavage activity induced by the injection of Cas9 RNPs into eggs. The *Dma-ey* gene was chosen as a target of Cas9 RNPs. For the preparation of the *Dma-ey*-targeting Cas9 RNPs, we purified Cas9 proteins as described previously [34] and used two different *Dma-ey* gRNAs named gRNA-1 and gRNA-2, which had been used previously in knock-out experiments using *Cas9* mRNAs (Fig 1) [25]. The gRNA-1 and gRNA-2 sequences target exons 8 and 10 of the *Dma-ey* gene, respectively (Fig 1). The biallelic mutation of the *Dma-ey* gene results in a deformation of the compound eye [25].

**Fig 2:**
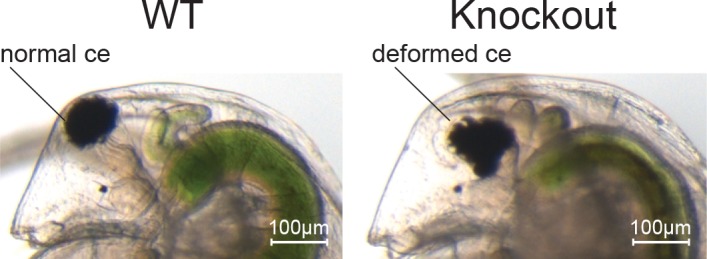
Phenotype of *Dma-ey* knock-out. The left image shows a normal compound eye (ce) in the wild type (WT) strain. The right image shows the *Dma-ey* knock-out phenotype with a deformed compound eye. The scale bar indicates 100 μm.

We first incubated 5 μM gRNA-1 with 2.5 μM Cas9 protein to generate Cas9 RNPs and injected them into 74 parthenogenetic female eggs (Table 1). At the first instar juvenile stage, 70% of the injected embryos survived. Of these, 83% showed deformed eye phenotypes (Table 1, Fig 2), suggesting that the injection of Cas9 RNPs led to somatic mutations. To calculate the efficiency of inducing heritable mutations, we counted the number of founder G0 animals that produced G1 progenies with deformed eyes. Of the surviving adults, 15% were founder G0 animals (Table 1). Second, we injected the same concentration of Cas9/gRNA-2 complex. The induction efficiencies of somatic and heritable mutations increased to 96% and 40%, which are two and five times higher than those achieved by the co-injection of *Cas9* mRNA together with gRNA-1 and gRNA-2 [25]. However, at the adult stage, the survival rate decreased to 30%. Therefore, we reduced the concentrations of gRNA and Cas9 protein by 2.5 times, which increased the survival rate to 57%. The heritable mutagenesis efficiency was approximately half of that induced by the co-injection of 2.5 μM Cas9 protein together with 5 μM gRNA-2, but it was still 1.4 times higher than that achieved by gRNA-1 injection (Table 1). These results demonstrated that the injection of Cas9 RNPs can lead to the highly efficient cleavage of a target site.

**Table 1:** Summary of knock-out experiments.

Concentration			Embryos	Juveniles		Adults	
gRNA		Cas9 protein	Injected	Surviving	Deformed eye	Surviving	Founder lines
#1	5 μM	2.5 μM	74	52/74 (70%)	43/52 (83%)	27/52 (52%)	4/27 (15%)
#2	5 μM	2.5 μM	82	45/82 (55%)	43/45 (96%)	25/82 (30%)	10/25 (40%)
	2 μM	1 μM	67	40/67 (60%)	26/40 (65%)	38/67 (57%)	8/38 (21%)

### Germline transmission of the reporter plasmid by the injection of Cas9 RNPs

**Fig 3:**
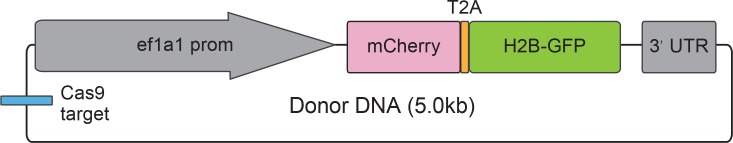
Structure of a donor plasmid for the knock-in at the *Dma-ey* locus. The *Thosea asigna* virus 2A (T2A) peptide connected the mCherry marker with a fusion protein comprising histone 2B (H2B) connected to green fluorescent protein. The *ef1a1* promoter/enhancer was inserted upstream from the T2A reporter cassette. The Cas9 target site (gRNA-2 recognition site) was derived from *Dma-ey* exon 10.

To test whether the injection of Cas9 RNPs can achieve the knock-in of plasmid DNA, we used the same *Dma-ey*-targeting gRNA-2 that had been used in the knock-out experiment (Fig 1) because it led to a higher efficiency of heritable mutagenesis than gRNA-1 (Table 1). To confirm the genomic integration and germline transmission of foreign DNA, we constructed a bicistronic fluorescent reporter plasmid encoding mCherry and a fusion protein comprising histone 2B (H2B) connected to green fluorescent protein, which were both linked via the self-cleaving 2A peptide from the *Thosea asigna* virus (T2A) (Fig 3). We used the *D*. *magna ef1a1* promoter/enhancer, which drives expression ubiquitously [13]. We inserted the gRNA-2 recognition site upstream from the *ef1a1* promoter/enhancer because the concurrent cleavage of genomic and plasmid DNA is known to increase the efficiency of integrating donor plasmid DNA into the genome [21].

**Fig 4:**
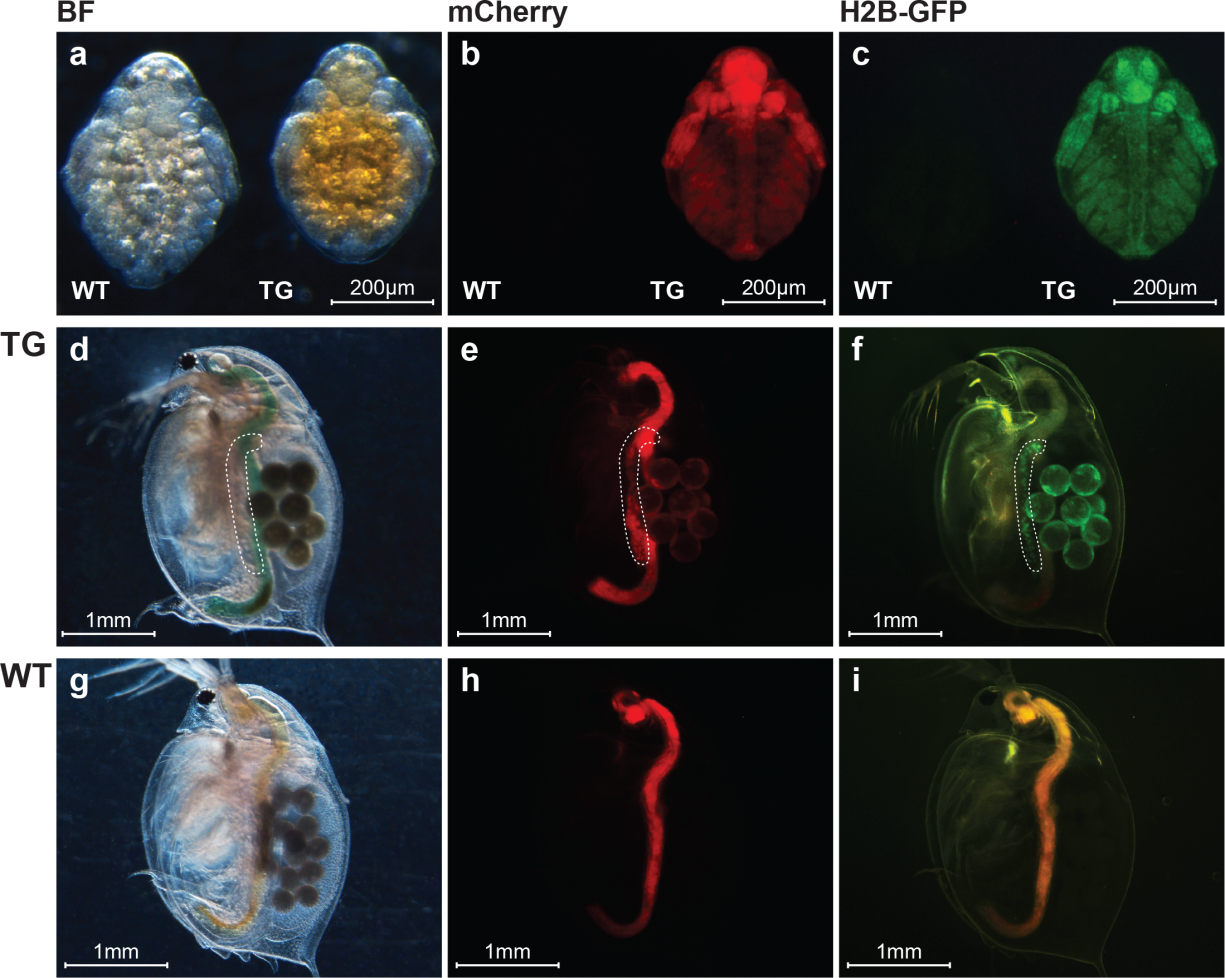
Phenotype of transgenic *D*. *magna*. The top row shows embryos obtained from wild type (WT) and transgenic (TG) lines. The middle and bottom rows show mature TG and WT *D*. *magna*, respectively. The images in each column were taken from the same individual under brightfield microscopy (left), with an mCherry filter (middle), and with a GFP2 filter (right). The regions surrounded by the white dashed lines are ovaries. The TG *D*. *magna* showed both red and green fluorescence. The mCherry fluorescence seen in the gut of the WT individuals is due to the autofluorescence of algae (chlorophyll has a fluorescence spectrum similar to that of mCherry and the gut is full of ingested algae). Under the GFP2 filter, which is a long pass filter with a peak transmission of 510 nm for emission, yellow autofluorescence could sometimes be detected, which was probably dependent on the gut contents as observed in the WT animals.

We co-injected 50 ng/μL donor plasmid DNA (the same concentration as used in the TALEN-mediated knock-in experiment [28]) together with Cas9 RNPs into *D*. *magna* eggs. To increase the survival rate, we used 1 μM Cas9 and 2.5 μM gRNAs (Table 1). Ninety-one adults survived out 203 injected eggs (45% survival, Table 2). Of these, 58 showed a deformed eye phenotype. One founder produced juveniles showing both red and green fluorescence, suggesting the successful germline transmission of the reporter plasmid (Table 2, Fig 4). This line also showed ubiquitous red and green fluorescence during the embryonic stages (Fig 4A–4C). However, in adults, both the red and green fluorescence became localized in the matured ovaries (Fig 4D–4I). We cultured this potential transgenic line and performed a genotyping analysis.

**Table 2:** Summary of knock-in experiment.

	Total number
Injected eggs	203
Adults	91/203 (45%)
Deformed eye	58/91 (64%)
Germline transmission	1 (1%)

### NHEJ-mediated knock-in of the reporter plasmid

**Fig 5:**
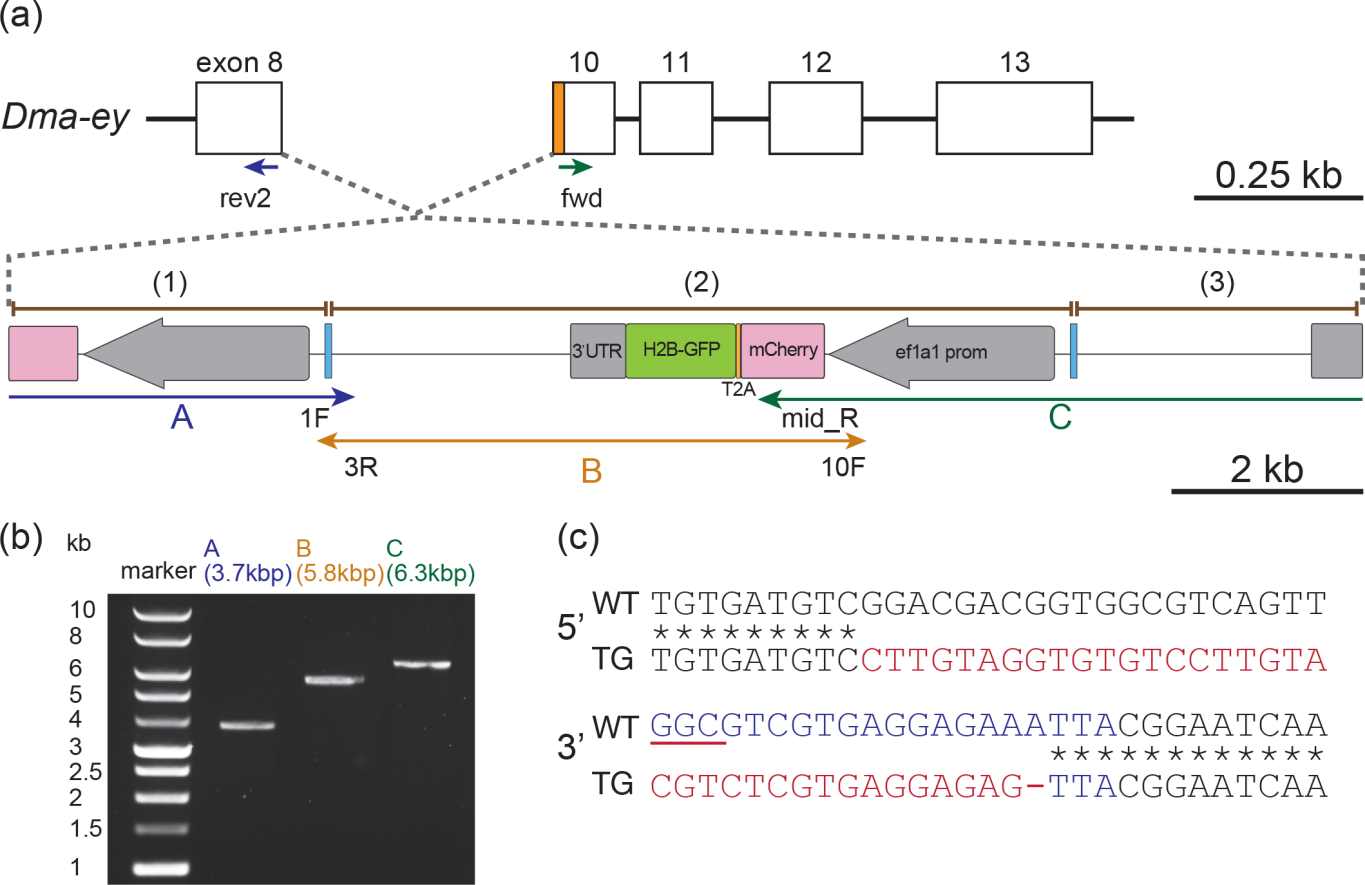
Characterization of the transgene structure. (a) Illustration of the endogenous locus where donor DNA was integrated. rev2, fwd, 1F, 3R, 10F, and mid_R indicate the positions of primers used for the amplification of the regions A, B, and C by polymerase chain reaction to determine the structure of the integrated donor DNA. The pink, orange, green, grey, and blue boxes and the grey arrows indicate the mCherry coding region, T2A region, H2B-GFP coding region, *ef1a1* 3′ UTR, Cas9 target region, and *ef1a1* promoter/enhancer region, respectively. (b) Polymerase chain reaction to determine the transgene structure. The amplified genomic DNA fragments were resolved by agarose gel electrophoresis. (c) The sequences of the junction regions between the transgene and the surrounding genome. The target sites for gRNA are indicated by blue letters and insertions are indicated by red letters. The protospacer adjacent motif sequence is underlined with red.

To confirm whether the donor plasmid DNA was inserted into the target site in the *Dma-ey* locus in the potentially transgenic *D*. *magna* line, the 5′ and 3′ junctions between the transgene and its surrounding genomic regions were amplified by polymerase chain reaction (PCR), cloned, and subsequently sequenced (Fig 5A and 5B, fragments A and C, S1 Fig). The 3′ junction was mapped to the target site in exon 10 and contained a 17-bp deletion and a 16-bp insertion, suggesting that NHEJ repair occurred at this site (Fig 5A and 5C). The 5′ junction was located in exon 8 and containing a 20-bp deletion and a 20-bp insertion (Fig 5C). These indels seemed to be larger than those introduced by TALEN-mediated knock-in [28]. The cleaved genome was ligated to mCherry and the 3′ untranslated region (UTR) sequences from the donor plasmid at the 5′ and 3′ junctions, respectively. Finally, the center of the integrated DNA was amplified by PCR and sequenced (Fig 5B, fragment B, S1 Fig). The sequences of the three PCR fragments were assembled into one unique sequence (S1 Fig) and compared with the donor plasmid sequence (S2 Fig). This revealed that the total length of the integrated transgenes was 13968 bp and three donor plasmids were tandemly integrated in reverse orientation at the target site. Except for the linearized plasmid in the center, the two plasmids at either end were abruptly terminated, showing large deletions. The Cas9 target sites between the integrated donor plasmids did not contain any mutation (S3 Fig). These results showed that the injection of Cas9 RNPs could lead to the knock-in of a plasmid via NHEJ.

## Discussion

We report that using the CRISPR/Cas system allowed us to knock-in a plasmid via NHEJ in *D*. *magna*. We previously showed that the co-injection of Cas9 mRNA together with two *eyeless*-targeting gRNAs (gRNA-1 and gRNA-2) led to the cleavage of a target locus and the introduction of indel mutations. Here, for the knock-in of a plasmid, the gRNA-2 that recognizes exon 10 of *Dma-ey* was mixed with Cas9 protein and the resulting Cas9 RNPs were co-injected together with a reporter plasmid that contains the gRNA-2 recognition site. Using this approach, we were able to successfully knock-in a reporter plasmid at the target site. Thus, we demonstrated that the CRISPR/Cas system can be applied not only for the knock-out but also for the knock-in of a gene of interest in *D*. *magna*.

In this study, we first tested the mutagenesis efficiency by the injection of Cas9 RNPs. When we used the *Dma-ey-*targeting gRNA-2, the somatic and heritable mutagenesis efficiencies were up to 96% and 40%, which are two and five times higher than those achieved by the co-injection of Cas9 mRNA together with gRNA-1 and gRNA-2 [25], even though a previous report suggested that the co-injection of multiple gRNAs increased the mutation efficiency [35]. This result suggests that the injection of Cas9 RNPs increases the genome-editing efficiency. The lack of a requirement for translation and the increased stability of gRNA in RNPs might enhance target cleavage [29–31,36]. Additionally, this method allows one to skip the laborious and time-consuming steps of preparing capped and polyadenylated mRNAs. We found that a higher concentration of Cas9 RNPs achieved a higher mutagenesis efficiency, but it decreased the survival rate of the injected embryos, probably due to having an off-target activity, and this phenomenon forced us to use a lower concentration of Cas9 RNPs for the knock-in experiments. As was reported recently, the utilization of chemically synthesized gRNAs could reduce the rate of off-target cleavage [37]. Given the high efficiency and simplicity of genome editing with Cas9 protein, the use of this method can significantly advance future studies on gene manipulation in *D*. *magna*.

In the knock-in experiment, the 3′ junction of the transgene was located at the Cas9 target site of the genome, which contained the protospacer adjacent motif sequence, demonstrating that the injected Cas9 RNPs cleaved the genome and NHEJ repair occurred at the target site (Fig 4). The 5′ and 3′ ends of the transgene lacked Cas9-target sites and contained a large deletion of the donor plasmid. In contrast, in the TALEN-mediated knock-in of a donor plasmid via NHEJ, we previously found that the 5′ and 3′ junctions had no indels or only small indels in all of the three transgenic *D*. *magna* lines that we generated [28]. This suggests that TALEN may achieve a more precise integration of the donor plasmid than CRISPR/Cas.

In the present study, the genotyping results indicated that the three plasmids were ligated at the Cas9 target site and were tandemly integrated into the *Dma-ey* locus. These results suggest that after the plasmid DNAs were injected into *D*. *magna*, they were cleaved by Cas9 at their target site, concatemerized, and digested by endogenous endonucleases in a sequence-independent manner, and the resulting concatemer was introduced into the Cas9 target site in the genome. Concatemeric plasmid DNA insertion has previously been found in random integration [13] and TALEN-mediated knock-in via NHEJ in *Daphnia* [28], suggesting that this type of plasmid insertion may be a common feature of knock-in via NHEJ in this species as well as some other organisms [21,28]. We also speculate that the concatemeric insertion might induce an unexpected effect of silencing the transgene in adults as reported in *Arabidopsis* [38], *Drosophila* [39], and mice [40].

We succeeded in establishing one transgenic line by the Cas9 RNPs injection method. The target site, which is located in exon 10 of the *Dma-ey* gene, was also used in the NHEJ-mediated knock-in via TALEN. The efficiency of genomic integration by Cas9 was approximately 1%, although we need to increase the number of injected eggs to further evaluate this efficiency. Still, this relatively low efficiency was consistent with that of TALEN-mediated knock-in (around 2–3%) [27,28]. These low knock-in efficiencies are possibly due to the nature of the *Dma-ey* gene, which is lethal to *D*. *magna* embryos when both alleles are mutated [25]. Given their abilities to mediate genetic knock-out, Cas9 RNPs and TALEN mRNAs might simultaneously introduce indel mutations in one allele while integrating plasmid DNA into the other allele, causing the death of the treated embryos. This suggests that we need to evaluate other loci to determine whether they may be more suitable for gene knock-in in *D*. *magna*.

In summary, the injection of Cas9 RNPs into *D*. *magna* eggs allowed NHEJ-mediated knock-in. This novel method, together with the technique of CRISPR/Cas-mediated knock-out, will enable us to analyze the link between genetic information and phenotypes in the model organism *Daphnia*.

## Materials and methods

### *D*. *magna* strain and culture conditions

The *D*. *magna* strain (NIES clone) was obtained from the National Institute for Environmental Studies (NIES; Tsukuba, Japan) and cultured under laboratory conditions for multiple generations as described previously [41] using Aachener Daphnien culture medium [42].

### Generation of donor plasmids

To generate the donor plasmid, we used the In-Fusion^®^ HD Cloning Kit (Clontech Laboratories, Inc., CA, USA) to clone the mCherry fragment, which contained the T2A sequence (5′-GAGGGCAGG GGAAGTCTT CTAACATGC GGGGACGTG GAGGAAAAT CCCGGGCCC-3′) at the 3′ end, upstream from the enhanced green fluorescent protein-fused *D*. *magna H2B* gene in pCS-EF1α1::H2B-GFP [13]. We amplified the Cas9 target site using *Daphnia* genomic DNA as template and inserted it upstream from the *ef1a1* promoter/enhancer region. The primer sequences for the amplification of the Cas9 target site were as follows: *ey2* forward primer (5′-ACAGGTTTGGTTCAGTAACCG-3′), *ey2* reverse primer (5′-CTGCTGCTGGGGATTGAC-3′).

### *In vitro* RNA synthesis

The gRNA expression vector pDR274-Dma-ey was generated as described previously [25]. To synthesize gRNAs, pDR274-Dma-ey vectors were digested using DraI and purified by phenol/chloroform extraction. The digested DNA fragments were used for *in vitro* transcription with the mMessage mMachine^®^ T7 kit (Life Technologies, Carlsbad, CA, USA). The synthesized RNAs were purified using mini Quick Spin^®^ RNA columns (Roche Diagnostics GmbH, Mannheim, Germany), phenol/chloroform extraction, and ethanol precipitation and were finally dissolved in DNase/RNase-free water.

### Microinjection

We mixed *in vitro* synthesized RNA with Cas9 protein to make gRNA-Cas9 complexes. Cas9 protein was prepared as described previously [34]. The gRNA-Cas9 complexes were incubated for 5 min at 37°C and injected into wild type *D*. *magna* eggs, as described previously [11]. The donor plasmids were mixed with the gRNA-Cas9 complexes before microinjection. In brief, we collected eggs from adult daphniids immediately after ovulation and stored them in ice-chilled M4 medium containing 80 mM sucrose (M4-sucrose). The injection volume was approximately 0.2 nL. We transferred the successfully injected eggs to fresh M4-sucrose and cultured them in a 96-well plate for 3 days at 23°C.

### Genomic DNA extraction and genotyping

For genomic DNA extraction, we homogenized a single daphniid using a Micro Smash homogenizer (TOMY, Tokyo, Japan) at 3,000 rpm for 1.5 min in 500 μL of lysis buffer (1.2% sodium dodecyl sulfate, 60 mM Tris-HCl, 24 mM ethylenediaminetetraacetic acid, and 1.2 M NaCl, pH 7.5). After homogenization, we added 7.5 μL of 10 mg/mL proteinase K solution (Nacalai Tesque, Kyoto, Japan) and 1 μL of salmon sperm DNA (10 mg/mL, Invitrogen, Carlsbad, CA, USA), and incubated the solution at 50°C overnight. We purified genomic DNA with phenol/chloroform and precipitated it with isopropanol. The pellet was washed with 70% ethanol and then dissolved in 50 μL TE buffer. We amplified the target region by PCR with PrimeSTAR^®^ GXL DNA polymerase (Takara Bio, Shiga, Japan). To amplify the plasmid-genomic DNA junctions (5′ and 3′ regions), we used the following primers: rev2 forward 5′-CTGCTGCTGGGGATTGAC-3′, 1F reverse 5′-GATTTAGAGCTTGACGGGGAAA-3′; 3R forward 5′-AACAAGGCGATAAAAGCAACG-3′, 10F reverse 5′-GGGCAATGGCTAAATCTTTCA-3′; mid_R forward 5′-ACCTTGAAGCGCATGAACTCC-3′, and fwd reverse 5′-CACCAAATCCGTTCATTGA -3′.

## Supporting information

S1 Fig Full sequence of the three tandemly integrated donor DNAs. Gray-shaded nucleotides indicate the *ef1a1* promoter/enhancer region. Purple, gray, pink, orange, green, and blue letters indicate *Dma-ey* and the *ef1a1* 3′ UTR, mCherry coding region, T2A region, H2B-GFP coding region, and Cas9 target region, respectively. Primers are indicated by bold type. Underlines show the indel mutation regions.
(DOCX)

S2 Fig Full sequence of donor DNA. Arrows indicate the positions of the primers used for genomic polymerase chain reaction.
(DOCX)

S3 Fig Comparison of the Cas9 target sites in the transgene region with the original sequence. WT shows the original *Dma-ey* targeting gRNA-2 sequence. TG 1st and TG 2nd show the first and second target sites of the transgene region in the same orientation as the endogenous *eyeless* gene. No mutation was found in either of the target sites.
(DOCX)
