# Supplementary material for: CRISPR/Cas-mediated knock-in via non-homologous end-joining in the crustacean Daphnia magna
Source: PLoS One. 2017 Oct 18;12(10):e0186112. doi: 10.1371/journal.pone.0186112 (PMC5646780; doi:10.1371/journal.pone.0186112)
Supplement: S1 Fig — Gray-shaded nucleotides indicate the ef1a1 promoter/enhancer region. Purple, gray, pink, orange, green, and blue letters indicate Dma-ey and the ef1a1 3′ UTR, mCherry coding region, T2A region, H2B-GFP coding region, and Cas9 target region, respectively. Primers are indicated by bold type. Underlines show the indel mutation regions. (DOCX) [file pone.0186112.s001.docx]

*eyeless* gene

*ef1a1* promoter/enhancer region

*ef1a1* 3′ UTR

mCherry coding region

T2A region

H2B-GFP coding region

Cas9 targeted region

Indel mutation region

5’-

gttcctttgatctttgttgtcgtctctcttaaatgggttactgtatctcagtgatttatgacgtagcaacaaatctcgcttggccaatcagacgttatct 100

**rev2**

taaaaacgaaaaaaaaaaaaaacatgattgttggtcctgtcttttcaaattggctttacacgtatttatgtatgtttttgttatctcattgtaggcggtg 200

acgatgtgatgtcCTTGTAGGTGTGTCCTTGTAGGCCTTGTAGGTGGTCTTGACCTCAGCGTCGTAGTGGCCGCCGTCCTTCAGCTTCAGCCTCTGCTTG 300

ATCTCGCCCTTCAGGGCGCCGTCCTCGGGGTACATCCGCTCGGAGGAGGCCTCCCAGCCCATGGTCTTCTTCTGCATTACGGGGCCGTCGGAGGGGAAGT 400

TGGTGCCGCGCAGCTTCACCTTGTAAATGAACTCGCCGTCCTGCAGGGAGGAGTCCTGGGTCACGGTCACCACGCCGCCGTCCTCGAAGTTCATCACGCG 500

CTCCCACTTGAAGCCCTCGGGGAAGGACAGCTTCAAGTAGTCGGGGATGTCGGCGGGGTGCTTCACGTAGGCCTTGGAGCCGTACATGAACTGAGGGGAC 600

AGGATGTCCCAGGCGAAGGGCAGGGGGCCACCCTTGGTCACCTTCAGCTTGGCGGTCTGGGTGCCCTCGTAGGGGCGGCCCTCGCCCTCGCCCTCGATCT 700

CGAACTCGTGGCCGTTCACGGAGCCCTCCATGTGCACCTTGAAGCGCATGAACTCCTTGATGATGGCCATGTTATCCTCCTCGCCCTTGCTCACCAGATC 800

CTCTTCTGAGATGAGTTTTTGTTCAAAGGTGTAGTCGGATTTCTTGTAGCCCCCAGTGGGATAGGCCAGTGCCAGTTCCTGATCCTGTCGCTTGCTGCAG 900

CATTTGTTGCCCATACCCGGGCATTGTGATTGGAGTTTAGGATCGGTTGAACAAACTGAAACAGCTGCAACAAAGAAAGAACTTTAAATTTCTTGAAGAC 1000

GAGCTGAACTCAACCGACCTACAAGGTAAAAGAGAATGAAAGATCCCTACAAAATTCACCCTATTTATATCGAATGACCACACCTCAGTAAAGTAAAACC 1100

ACTTGAGGGCAACTAACCCCACACCCAGTAACCACCAGTTTATTAACCCCAACTGAAATTCGCCCCCACGACCTTGAACTAAAAAGATTAATGTTAAATG 1200

ATTTTCAAGGGCAAATTATTCAAAAGAATTAGTGAAAATGTATGAAAGATTTAGCCATTGCCCAATTGGGACTACACCCCGGTAAATAAGTGGAGGCGCC 1300

ACAGCATGAAGAAATACTGAAATCCTACAAAATGTCTAGAATGAAACCATGAAACTATTAATTCAATGTAATGAATGTTAACTTGTCCCTAGATTAGAAC 1400

TGAAAAGTACGAAAACGAAAATCGCCCCGCAATGGCGACCAAGCCCCACATGTCGATTGAAACCGGCCCACTTGACAGTTGCACTGGAAACTTTTCTCTA 1500

CAAAAAAACCCATCACCTTCTCATCGTTATTATCCTAAAATAGCTTAATTAAACGAAGAATGAGTATTTCTATGCTTTGAAGACATTGTTACTAATGGAT 1600

TGACAAAGAATACTTCGATTTGCGAGGATAAATTTGGGAGACGCGTCAAAACGTGTGCTTACCTGATGTTCCGTTGGAAGAGAAGAGAACGAACCTTGAA 1700

AGACACGAGAACGAGTGACGCTGGAAGTTGCTAGCCCCTACGGAGTAAAGTCTCTACAGGGGAGGGGAACAGAGTTGCATTGCGTTAAACATTGTTTAAT 1800

TTTATAACATTTCAATTGATTAATGTTAGAAAAATGTAGATTATTAATTTTTGATTTTATCAAACAACCACAATCTATTTACTGATGCTTCTTTCAAGGT 1900

TATTTAAATTTTTTTCTCCCTCGGATATTTCTCCCCCTCTCTAGGGGCTTTGCACAAGGTTGTTTTCAAGGTAAAAAAAAAAAACACATTGTTCCCAAAT 2000

GAATTTTTGATTTTAAAATTTTTATCTTAACAACTATAAAGTTTAACGTTGTTTGAAAATGTTTTCTTGCAAGTCTGATTTGTCGATTTGACATCTCTTT 2100

GTTTACTCTTCAGAACAAAATGGCCATCACGACTACCATGAGCAAATTGTTAAATTTTTCAAGTTTTTTATTGCAAAACACCGCCCAACTACTTCGAAAT 2200

GGAGTATGTTTTTATATTTTTCGTTTTATATATCAGTTACGTAAACAAAACAAAATTTCTTAGATGACAAATGCAGTAAAATTGAATCAACCAGTCAGAA 2300

ACAATTGGACAGATTGGAGAATGATGAAGGATAACCGGCGACGAAAATTACTCGTCCAATACGCACCTGACCGTGTTCGCGCAAAAGCCCTAAAAAAAAA 2400

TGATGTTTTGCCTGCTGAGATAATAGTAATTTAGAACTACCCTTCATTCTTAGTTGAACTAATTCTCTCACAGGCCTATTAAGTTATTTACCCAATATCG 2500

AGCTTTAACAAGTTTTGCATGGGATTTCACACTATTTTTTCATTAATTTTGTTGCGTTGGGTTTGCACTGTTGTTGAATTTTTATATAAATTTTTTAAAA 2600

TATTAAGATCAATTCATGCAATCATAGGATAAGCATCAGTATATTCTTTAAAGTATACAACAAGTTTAAGAACCCCTCATTTAAAGATTTTGTTTGAAGG 2700

AGATTGGTACCAAAATGCTAGCTGAAATTCCTCGAGATGCGAGCTTAACCCGCGTACGCAATCGCTGTGCCATCACGTCCCGTCCAAGAGGCGTCGTCAC 2800

GCGCTGGAGATTGAGTCGAATTGTTTGGCGTAGCTTGGCCGATTACAACAAACTGTCTGGAGTTCAACGAGCAATGTGGTGATCACGCATGTATTTCTAG 2900

AAAACAACCAATAAATTTCTCAATAGTCGTTGTATTGCTCCTTACATCACTTCCTGTATGAAATGACAATTACTTAGTGTAAATATTTACAAGCCAGAGA 3000

TGTTCTATGTAACTATAAGCCACTCTACATCTTGTAAGTATTGCATTGTTTTCCCAGTATAAAGGAAGGGCACAGCTCCACGATTGTCGATCAATCTTAC 3100

CAAATGACTGAAAGGAACGGCCCAGGGTGTAACTGGCTACAATTTGAAACAAGGCGATAAAAGCAACGTTACCTTTAAATTTAATCAATAGCTGTGTGAG 3200

**3R**

TTGAAAATTTTCCCCAAGGTCGCTGAAGCTTATAACTTCGTATAGCATACATTATACGAAGTTATGTCGACCTCGAAGCCGCGGTGCGGGTGCCAGGGCG 3300

TGCCCTTGGGCTCCCCGGGCGCGTACTCCACCTCACCCATCCTGCTGCTGGGGATTGACTTGATCCGGCTGTTGCGACGACGATTGTTGTTGTTCTGGTC 3400

CACGCCGTTGATTCCGTAATTTCTCCTCACGACGCCATTTTGCTCGTCGGTTACTGAACCAAACCTGTGTTGCGCAGCCTGAATGGCGAATGGGACGCGC 3500

CCTGTAGCGGCGCATTAAGCGCGGCGGGTGTGGTGGTTACGCGCAGCGTGACCGCTACACTTGCCAGCGCCCTAGCGCCCGCTCCTTTCGCTTTCTTCCC 3600

TTCCTTTCTCGCCACGTTCGCCGGCTTTCCCCGTCAAGCTCTAAATCGGGGGCTCCCTTTAGGGTTCCGATTTAGTGCTTTACGGCACCTCGACCCCAAA 3700

**1F**

AAACTTGATTAGGGTGATGGTTCACGTAGTGGGCCATCGCCCTGATAGACGGTTTTTCGCCCTTTGACGTTGGAGTCCACGTTCTTTAATAGTGGACTCT 3800

TGTTCCAAACTGGAACAACACTCAACCCTATCTCGGTCTATTCTTTTGATTTATAAGGGATTTTGCCGATTTCGGCCTATTGGTTAAAAAATGAGCTGAT 3900

TTAACAAAAATTTAACGCGAATTTTAACAAAATATTAACGCTTACAATTTAGGTGGCACTTTTCGGGGAAATGTGCGCGGAACCCCTATTTGTTTATTTT 4000

TCTAAATACATTCAAATATGTATCCGCTCATGAGACAATAACCCTGATAAATGCTTCAATAATATTGAAAAAGGAAGAGTATGAGTATTCAACATTTCCG 4100

TGTCGCCCTTATTCCCTTTTTTGCGGCATTTTGCCTTCCTGTTTTTGCTCACCCAGAAACGCTGGTGAAAGTAAAAGATGCTGAAGATCAGTTGGGTGCA 4200

CGAGTGGGTTACATCGAACTGGATCTCAACAGCGGTAAGATCCTTGAGAGTTTTCGCCCCGAAGAACGTTTTCCAATGATGAGCACTTTTAAAGTTCTGC 4300

TATGTGGCGCGGTATTATCCCGTATTGACGCCGGGCAAGAGCAACTCGGTCGCCGCATACACTATTCTCAGAATGACTTGGTTGAGTACTCACCAGTCAC 4400

AGAAAAGCATCTTACGGATGGCATGACAGTAAGAGAATTATGCAGTGCTGCCATAACCATGAGTGATAACACTGCGGCCAACTTACTTCTGACAACGATC 4500

GGAGGACCGAAGGAGCTAACCGCTTTTTTGCACAACATGGGGGATCATGTAACTCGCCTTGATCGTTGGGAACCGGAGCTGAATGAAGCCATACCAAACG 4600

ACGAGCGTGACACCACGATGCCTGTAGCAATGGCAACAACGTTGCGCAAACTATTAACTGGCGAACTACTTACTCTAGCTTCCCGGCAACAATTAATAGA 4700

CTGGATGGAGGCGGATAAAGTTGCAGGACCACTTCTGCGCTCGGCCCTTCCGGCTGGCTGGTTTATTGCTGATAAATCTGGAGCCGGTGAGCGTGGGTCT 4800

CGCGGTATCATTGCAGCACTGGGGCCAGATGGTAAGCCCTCCCGTATCGTAGTTATCTACACGACGGGGAGTCAGGCAACTATGGATGAACGAAATAGAC 4900

AGATCGCTGAGATAGGTGCCTCACTGATTAAGCATTGGTAACTGTCAGACCAAGTTTACTCATATATACTTTAGATTGATTTAAAACTTCATTTTTAATT 5000

TAAAAGGATCTAGGTGAAGATCCTTTTTGATAATCTCATGACCAAAATCCCTTAACGTGAGTTTTCGTTCCACTGAGCGTCAGACCCCGTAGAAAAGATC 5100

AAAGGATCTTCTTGAGATCCTTTTTTTCTGCGCGTAATCTGCTGCTTGCAAACAAAAAAACCACCGCTACCAGCGGTGGTTTGTTTGCCGGATCAAGAGC 5200

TACCAACTCTTTTTCCGAAGGTAACTGGCTTCAGCAGAGCGCAGATACCAAATACTGTCCTTCTAGTGTAGCCGTAGTTAGGCCACCACTTCAAGAACTC 5300

TGTAGCACCGCCTACATACCTCGCTCTGCTAATCCTGTTACCAGTGGCTGCTGCCAGTGGCGATAAGTCGTGTCTTACCGGGTTGGACTCAAGACGATAG 5400

TTACCGGATAAGGCGCAGCGGTCGGGCTGAACGGGGGGTTCGTGCACACAGCCCAGCTTGGAGCGAACGACCTACACCGAACTGAGATACCTACAGCGTG 5500

AGCTATGAGAAAGCGCCACGCTTCCCGAAGGGAGAAAGGCGGACAGGTATCCGGTAAGCGGCAGGGTCGGAACAGGAGAGCGCACGAGGGAGCTTCCAGG 5600

GGGAAACGCCTGGTATCTTTATAGTCCTGTCGGGTTTCGCCACCTCTGACTTGAGCGTCGATTTTTGTGATGCTCGTCAGGGGGGCGGAGCCTATGGAAA 5700

AACGCCAGCAACGCGGCCTTTTTACGGTTCGTGGAATTGTGAGCGGATAACAATTTCACACAGGAAACAGCTATGACCATGATTACGCCAAGCGCGCAAT 5800

TAACGAGGCTCTCGATCTGTTCGTTGGTGAAGGAAGTGCGGTTGCGCTGGAGTTTGCGCTTGAGTCGCAGTCGAGCCTGATCGTCGTCCGAAGGTGTTGT 5900

TGTCGTCGGCGTGGGCGGAGCCGACCGAGAATTCTCGCCGGAACTGACGCCACCGTCGTCCGACATCACATCGTCACAAGGGAACAAAAGCTGGGTACCG 6000

GGGTCCAAATTATCTTGTATTGGAGCTTGATTGAGGTGCATATACCAAAGCCCTTACACATACAGGGAGTAAGTAAAAACGATTCGAATAAACCAAGTGG 6100

AAAGTTAACAATTTACCAATAAAGCCACCTCAATAATGTACAAGATGACTAATAAAAGTAAAATGCACTCAGAAAGTACACGAGGGCGCTGTTTTTCAAT 6200

TAATCAAACCAAGATATCATTATTCTTCTCCAGGATTACCCACTCCAATAGTAGGAAACGCGCTAAAATTTCGTGTTAAAGGAAGAAAATACGACACAAA 6300

TTCAACAGGATACAAGCAAAGACATACTGTAGACAGATAGTCTAGTCCTACATAACAAAGTTGAAAACAAGTTGCTTCAACCAAACGCTTGCTTTTCTCC 6400

TGAATTCTTTTCTATGCCGATTGAAGAATTTTTTTACGGAAACTAAATGAAGAATTTCAAACTGTAGATGCTTCGGACCCGGTACATGACACTCTTGTCG 6500

GTTGGATGGAATAGTAGCCTCCATCTACTTGTCGTCATCGTCTTTGTAGTCCTTGTACAGCTCGTCCATGCCGAGAGTGATCCCGGCGGCGGTCACGAAC 6600

TCCAGCAGGACCATGTGATCGCGCTTCTCGTTGGGGTCTTTGCTCAGGGCGGACTGGGTGCTCAGGTAGTGGTTGTCGGGCAGCAGCACGGGGCCGTCGC 6700

CGATGGGGGTGTTCTGCTGGTAGTGGTCGGCGAGCTGCACGCTGCCGTCCTCGATGTTGTGGCGGATCTTGAAGTTCACCTTGATGCCGTTCTTCTGCTT 6800

GTCGGCCATGATATAGACGTTGTGGCTGTTGTAGTTGTACTCCAGCTTGTGCCCCAGGATGTTGCCGTCCTCCTTGAAGTCGATGCCCTTCAGCTCGATG 6900

CGGTTCACCAGGGTGTCGCCCTCGAACTTCACCTCGGCGCGGGTCTTGTAGTTGCCGTCGTCCTTGAAGAAGATGGTGCGCTCCTGGACGTAGCCTTCGG 7000

GCATGGCGGACTTGAAGAAGTCGTGCTGCTTCATGTGGTCGGGGTAGCGGCTGAAGCACTGCACGCCGTAGGTCAGGGTGGTCACGAGGGTGGGCCAGGG 7100

CACGGGCAGCTTGCCGGTGGTGCAGATGAACTTCAGGGTCAGCTTGCCGTAGGTGGCATCGCCCTCGCCCTCGCCGGACACGCTGAACTTGTGGCCGTTT 7200

ACGTCGCCGTCCAGCTCGACCAGGATGGGCACCACCCCGGTGAACAGCTCCTCGCCCTTGCTCACCATGGTGGCGACCGGTGGATCCTTTGTGCTGGTAT 7300

ACTTGGTGACAGCTTTGGTGCCTTCAGAGACGGCGTGTTTCGCTAATTCACCGGGCAAAAGAAGACGGACAGCTGTTTGGATTTCTCGACTCGTGATGGT 7400

AGAACGCTTGTTGTAGTGAGCAAGACGAGAGGATTCTCCAGCAATGCGCTCGAAAATATCGTTGACGAAGCTGTTCATGATCGTCATGGCTTTCGAGGAA 7500

ATACCAGTGTCGGGATGGACTTGCTTCAGCACTTTGTAGATGTAAATTGCGTAGCTCTCCTTCCTTCTACGCTTCTTCTTTTTATCGCCTTTAGTGATAT 7600

TCTTCTGAGCTTTCCCAGCTTTCTTCGCTGCTTTCCCACTTACTTTAGGGGGGTGATGGTGATGGTGATGGGGCCCGGGATTTTCCTCCACGTCCCCGCA 7700

TGTTAGAAGACTTCCCCTGCCCTCAGCGTAATCTGGAACATCGTATGGGTACTTGTACAGCTCGTCCATGCCGCCGGTGGAGTGGCGGCCCTCGGCGCGT 7800

TCGTACTGTTCCACGATGGTGTAGTCCTCGTTGTGGGAGGTGATGTCCAACTTGATGTTGACGTTGTAGGCGCCGGGCAGCTGCACGGGCTTCTTGGCCT 7900

TGTAGGTGGTCTTGACCTCAGCGTCGTAGTGGCCGCCGTCCTTCAGCTTCAGCCTCTGCTTGATCTCGCCCTTCAGGGCGCCGTCCTCGGGGTACATCCG 8000

CTCGGAGGAGGCCTCCCAGCCCATGGTCTTCTTCTGCATTACGGGGCCGTCGGAGGGGAAGTTGGTGCCGCGCAGCTTCACCTTGTAGATGAACTCGCCG 8100

TCCTGCAGGGAGGAGTCCTGGGTCACGGTCACCACGCCGCCGTCCTCGAAGTTCATCACGCGCTCCCACTTGAAGCCCTCGGGGAAGGACAGCTTCAAGT 8200

AGTCGGGGATGTCGGCGGGGTGCTTCACGTAGGCCTTGGAGCCGTACATGAACTGAGGGGACAGGATGTCCCAGGCGAAGGGCAGGGGGCCACCCTTGGT 8300

CACCTTCAGCTTGGCGGTCTGGGTGCCCTCGTAGGGGCGGCCCTCGCCCTCGCCCTCGATCTCGAACTCGTGGCCGTTCACGGAGCCCTCCATGTGCACC 8400

TTGAAGCGCATGAACTCCTTGATGATGGCCATGTTATCCTCCTCGCCCTTGCTCACCAGATCCTCTTCTGAGATGAGTTTTTGTTCAAAGGTGTAGTCGG 8500

**mid_R**

ATTTCTTGTAGCCCCCAGTGGGATAGGCCAGTGCCAGTTCCTGATCCTGTCGCTTGCTGCAGCATTTGTTGCCCATACCCGGGCATTGTGATTGGAGTTT 8600

AGGATCGGTTGAACAAACTGAAACAGCTGCAACAAAGAAAGAACTTTAAATTTCTTGAAGACGAGCTGAACTCAACCGACCTACAAGGTAAAAGAGAATG 8700

AAAGATCCCTACAAAATTCACCCTATTTATATCGAATGACCACACCTCAGTAAAGTAAAACCACTTGAGGGCAACTAACCCCACACCCAGTAACCACCAG 8800

TTTATTAACCCCAACTGAAATTCGCCCCCACGACCTTGAACTAAAAAGATTAATGTTAAATGATTTTCAAGGGCAAATTATTCAAAAGAATTAGTGAAAA 8900

TGTATGAAAGATTTAGCCATTGCCCAATTGGGACTACACCCCGGTAAATAAGTGGAGGCGCCACAGCATGAAGAAATACTGAAATCCTACAAAATGTCTA 9000

**10F**

GAATGAAACCATGAAACTATTAATTCAATGTAATGAATGTTAACTTGTCCCTAGATTAGAACTGAAAAGTACGAAAACGAAAATCGCCCCGCAATGGCGA 9100

CCAAGCCCCACATGTCGATTGAAACCGGCCCACTTGACAGTTGCACTGGAAACTTTTCTCTACAAAAAAACCCATCACCTTCTCATCGTTATTATCCTAA 9200

AATAGCTTAATTAAACGAAGAATGAGTATTTCTATGCTTTGAAGACATTGTTACTAATGGATTGACAAAGAATACTTCGATTTGCGAGGATAAATTTGGG 9300

AGACGCGTCAAAACGTGTGCTTACCTGATGTTCCGTTGGAAGAGAAGAGAACGAACCTTGAAAGACACGAGAACGAGTGACGCTGGAAGTTGCTAGCCCC 9400

TACGGAGTAAAGTCTCTACAGGGGAGGGGAACAGAGTTGCATTGCGTTAAACATTGTTTAATTTTATAACATTTCAATTGATTAATGTTAGAAAAATGTA 9500

GATTATTAATTTTTGATTTTATCAAACAACCACAATCTATTTACTGATGCTTCTTTCAAGGTTATTTAAATTTTTTTCTCCCTCGGATATTTCTCCCCCT 9600

CTCTAGGGGCTTTGCACAAGGTTGTTTTCAAGGTAAAAAAAAAAAACACATTGTTCCCAAATGAATTTTTGATTTTAAAATTTTTATCTTAACAACTATA 9700

AAGTTTAACGTTGTTTGAAAATGTTTTCTTGCAAGTCTGATTTGTCGATTTGACATCTCTTTGTTTACTCTTCAGAACAAAATGGCCATCACGACTACCA 9800

TGAGCAAATTGTTAAATTTTTCAAGTTTTTTATTGCAAAACACCGCCCAACTACTTCGAAATGGAGTATGTTTTTATATTTTTCGTTTTATATATCAGTT 9900

ACGTAAACAAAACAAAATTTCTTAGATGACAAATGCAGTAAAATTGAATCAACCAGTCAGAAACAATTGGACAGATTGGAGAATGATGAAGGATAACCGG 10000

CGACGAAAATTACTCGTCCAATACGCACCTGACCGTGTTCGCGCAAAAGCCCTAAAAAAAAATGATGTTTTGCCTGCTGAGATAATAGTAATTTAGAACT 10100

ACCCTTCATTCTTAGTTGAACTAATTCTCTCACAGGCCTATTAAGTTATTTACCCAATATCGAGCTTTAACAAGTTTTGCATGGGATTTCACACTATTTT 10200

TTCATTAATTTTGTTGCGTTGGGTTTGCACTGTTGTTGAATTTTTATATAAATTTTTTAAAATATTAAGATCAATTCATGCAATCATAGGATAAGCATCA 10300

GTATATTCTTTAAAGTATACAACAAGTTTAAGAACCCCTCATTTAAAGATTTTGTTTGAAGGAGATTGGTACCAAAATGCTAGCTGAAATTCCTCGAGAT 10400

GCGAGCTTAACCCGCGTACGCAATCGCTGTGCCATCACGTCCCGTCCAAGAGGCGTCGTCACGCGCTGGAGATTGAGTCGAATTGTTTGGCGTAGCTTGG 10500

CCGATTACAACAAACTGTCTGGAGTTCAACGAGCAATGTGGTGATCACGCATGTATTTCTAGAAAACAACCAATAAATTTCTCAATAGTCGTTGTATTGC 10600

TCCTTACATCACTTCCTGTATGAAATGACAATTACTTAGTGTAAATATTTACAAGCCAGAGATGTTCTATGTAACTATAAGCCACTCTACATCTTGTAAG 10700

TATTGCATTGTTTTCCCAGTATAAAGGAAGGGCACAGCTCCACGATTGTCGATCAATCTTACCAAATGACTGAAAGGAACGGCCCAGGGTGTAACTGGCT 10800

ACAATTTGAAACAAGGCGATAAAAGCAACGTTACCTTTAAATTTAATCAATAGCTGTGTGAGTTGAAAATTTTCCCCAAGGTCGCTGAAGCTTATAACTT 10900

CGTATAGCATACATTATACGAAGTTATGTCGACCTCGAAGCCGCGGTGCGGGTGCCAGGGCGTGCCCTTGGGCTCCCCGGGCGCGTACTCCACCTCACCC 11000

ATCCTGCTGCTGGGGATTGACTTGATCCGGCTGTTGCGACGACGATTGTTGTTGTTCTGGTCCACGCCGTTGATTCCGTAATTTCTCCTCACGACGCCAT 11100

TTTGCTCGTCGGTTACTGAACCAAACCTGTGTTGCGCAGCCTGAATGGCGAATGGGCTGAATGGCGAATGGGACGCGCCCTGTAGCGGCGCATTAAGCGC 11200

GGCGGGTGTGGTGGTTACGCGCAGCGTGACCGCTACACTTGCCAGCGCCCTAGCGCCCGCTCCTTTCGCTTTCTTCCCTTCCTTTCTCGCCACGTTCGCC 11300

GGCTTTCCCCGTCAAGCTCTAAATCGGGGGCTCCCTTTAGGGTTCCGATTTAGTGCTTTACGGCACCTCGACCCCAAAAAACTTGATTAGGGTGATGGTT 11400

CACGTAGTGGGCCATCGCCCTGATAGACGGTTTTTCGCCCTTTGACGTTGGAGTCCACGTTCTTTAATAGTGGACTCTTGTTCCAAACTGGAACAACACT 11500

CAACCCTATCTCGGTCTATTCTTTTGATTTATAAGGGATTTTGCCGATTTCGGCCTATTGGTTAAAAAATGAGCTGATTTAACAAAAATTTAACGCGAAT 11600

TTTAACAAAATATTAACGCTTACAATTTAGGTGGCACTTTTCGGGGAAATGTGCGCGGAACCCCTATTTGTTTATTTTTCTAAATACATTCAAATATGTA 11700

TCCGCTCATGAGACAATAACCCTGATAAATGCTTCAATAATATTGAAAAAGGAAGAGTATGAGTATTCAACATTTCCGTGTCGCCCTTATTCCCTTTTTT 11800

GCGGCATTTTGCCTTCCTGTTTTTGCTCACCCAGAAACGCTGGTGAAAGTAAAAGATGCTGAAGATCAGTTGGGTGCACGAGTGGGTTACATCGAACTGG 11900

ATCTCAACAGCGGTAAGATCCTTGAGAGTTTTCGCCCCGAAGAACGTTTTCCAATGATGAGCACTTTTAAAGTTCTGCTATGTGGCGCGGTATTATCCCG 12000

TATTGACGCCGGGCAAGAGCAACTCGGTCGCCGCATACACTATTCTCAGAATGACTTGGTTGAGTACTCACCAGTCACAGAAAAGCATCTTACGGATGGC 12100

ATGACAGTAAGAGAATTATGCAGTGCTGCCATAACCATGAGTGATAACACTGCGGCCAACTTACTTCTGACAACGATCGGAGGACCGAAGGAGCTAACCG 12200

CTTTTTTGCACAACATGGGGGATCATGTAACTCGCCTTGATCGTTGGGAACCGGAGCTGAATGAAGCCATACCAAACGACGAGCGTGACACCACGATGCC 12300

TGTAGCAATGGCAACAACGTTGCGCAAACTATTAACTGGCGAACTACTTACTCTAGCTTCCCGGCAACAATTAATAGACTGGATGGAGGCGGATAAAGTT 12400

GCAGGACCACTTCTGCGCTCGGCCCTTCCGGCTGGCTGGTTTATTGCTGATAAATCTGGAGCCGGTGAGCGTGGGTCTCGCGGTATCATTGCAGCACTGG 12500

GGCCAGATGGTAAGCCCTCCCGTATCGTAGTTATCTACACGACGGGGAGTCAGGCAACTATGGATGAACGAAATAGACAGATCGCTGAGATAGGTGCCTC 12600

ACTGATTAAGCATTGGTAACTGTCAGACCAAGTTTACTCATATATACTTTAGATTGATTTAAAACTTCATTTTTAATTTAAAAGGATCTAGGTGAAGATC 12700

CTTTTTGATAATCTCATGACCAAAATCCCTTAACGTGAGTTTTCGTTCCACTGAGCGTCAGACCCCGTAGAAAAGATCAAAGGATCTTCTTGAGATCCTT 12800

TTTTTCTGCGCGTAATCTGCTGCTTGCAAACAAAAAAACCACCGCTACCAGCGGTGGTTTGTTTGCCGGATCAAGAGCTACCAACTCTTTTTCCGAAGGT 12900

AACTGGCTTCAGCAGAGCGCAGATACCAAATACTGTCCTTCTAGTGTAGCCGTAGTTAGGCCACCACTTCAAGAACTCTGTAGCACCGCCTACATACCTC 13000

GCTCTGCTAATCCTGTTACCAGTGGCTGCTGCCAGTGGCGATAAGTCGTGTCTTACCGGGTTGGACTCAAGACGATAGTTACCGGATAAGGCGCAGCGGT 13100

CGGGCTGAACGGGGGGTTCGTGCACACAGCCCAGCTTGGAGCGAACGACCTACACCGAACTGAGATACCTACAGCGTGAGCTATGAGAAAGCGCCACGCT 13200

TCCCGAAGGGAGAAAGGCGGACAGGTATCCGGTAAGCGGCAGGGTCGGAACAGGAGAGCGCACGAGGGAGCTTCCAGGGGGAAACGCCTGGTATCTTTAT 13300

AGTCCTGTCGGGTTTCGCCACCTCTGACTTGAGCGTCGATTTTTGTGATGCTCGTCAGGGGGGCGGAGCCTATGGAAAAACGCCAGCAACGCGGCCTTTT 13400

TACGGTTCGTGGAATTGTGAGCGGATAACAATTTCACACAGGAAACAGCTATGACCATGATTACGCCAAGCGCGCAATTAACGAGGCTCTCGATCTGTTC 13500

GTTGGTGAAGGAAGTGCGGTTGCGCTGGAGTTTGCGCTTGAGTCGCAGTCGAGCCTGATCGTCGTCCGAAGGTGTTGTTGTCGTCGGCGTGGGCGGAGCC 13600

GACCGAGAATTCTCGCCGGAACTGACGCCACCGTCGTCCGACATCACATCGTCACAAGGGAACAAAAGCTGGGTACCGGGGTCCAAATTATCTTGTATTG 13700

GAGCTTGATTGAGGTGCATATACCAAAGCCCTTACACATACAGGGAGTAAGTAAAAACGATTCGAATAAACCAAGTGGAAAGTTAACAATTTACCAATAA 13800

AGCCACCTCAATAATGTACAAGATGACTAATAAAAGTAAAATGCACTCAGAAAGTACACGAGGGCGCTGTTTTTCAATTAATCAAACCAAGATATCATTA 13900

TTCTTCTCCAGGATTACCCACTCCAATAGTAGGAAACGCGCTAAAATTTCGTGTTAAAGGAAGAAAATACGACACAAATTCAACAGGATACAAGCAAAGA 14000

CATACTGTAGACAGATAGTCTAGTCCTACATAACAAAGTTGAAAACAAGTTGCTTCAACCAAACGCTTGCTTTTCTCCTGAATTCTTTTCTATGCCGATT 14100

GAAGAATTTTTTTACGGAAACTAAATGAAGAATTTCAAACTGTAGATGCTTCGGACCCGGTACATGACACTCTTGTCGGTTGGATGGAATAGTAGCCTCC 14200

Acgtctcgtgaggagagttacggaatcaacggcgtggaccagaacaacaacaatcgtcgtcgcaacagccggatcaagtcaatccccagcagcagcaaca 14300

acaacagcaacaacagcagctgatccctgtggttgaacctgcacccagttcgccccgcagtgtttctcattccgccaattatcccagtggcatttattca 14400

tctttggccactaccatgatgactgatacatacaggtaatcaatgaatttaaatttgactctttattcagcagctgatccctgtggttgaacctgcaccc 14500

agttcgccccgcagtgtttctcattccgccaattatcccagtggcatttattcatctttggccactaccatgatgactgatacatacaggtaatcaatga 14600

atttaaatttgactctttattccgcgcggtttttaacggtttgcgttccccgtgtttatctaaatcctttttttagttccgcgtttaatggaatgaattc 14700

**fwd**

aatgaacggatttggtg -3’

**S1 Fig. Full sequence of the three tandemly integrated donor DNAs.** Gray-shaded nucleotides indicate the *ef1a1* promoter/enhancer region. Purple, gray, pink, orange, green, and blue letters indicate *Dma-ey* and the *ef1a1* 3′ UTR, mCherry coding region, T2A region, H2B-GFP coding region, and Cas9 target region, respectively. Primers are indicated by bold type. Underlines show the indel mutation regions.
