# Supplementary material for: CRISPR/Cas-mediated knock-in via non-homologous end-joining in the crustacean Daphnia magna
Source: PLoS One. 2017 Oct 18;12(10):e0186112. doi: 10.1371/journal.pone.0186112 (PMC5646780; doi:10.1371/journal.pone.0186112)
Supplement: S2 Fig — Arrows indicate the positions of the primers used for genomic polymerase chain reaction. (DOCX) [file pone.0186112.s002.docx]

*ef1a1* promoter/enhancer region

*ef1a1* 3′ UTR

mCherry coding region

T2A region

H2B-GFP coding region

Cas9 targeted region

5’-

AGCTTCAGCGACCTTGGGGAAAATTTTCAACTCACACAGCTATTGATTAAATTTAAAGGTAACGTTGCTTTTATCGCCTTGTTCAAATTGTAGCCAGTT 100

**3R**

ACACCCTGGGCCGTTCCTTTCAGTCATTTGGTAAGATTGATCGACAATCGTGGAGCTGTGCCCTTCCTTTATACTGGGAAAACAATGCAATACTTACAAG 200

ATGTAGAGTGGCTTATAGTTACATAGAACATCTCTGGCTTGTAAATATTTACACTAAGTAATTGTCATTTCATACAGGAAGTGATGTAAGGAGCAATACA 300

ACGACTATTGAGAAATTTATTGGTTGTTTTCTAGAAATACATGCGTGATCACCACATTGCTCGTTGAACTCCAGACAGTTTGTTGTAATCGGCCAAGCTA 400

CGCCAAACAATTCGACTCAATCTCCAGCGCGTGACGACGCCTCTTGGACGGGACGTGATGGCACAGCGATTGCGTACGCGGGTTAAGCTCGCATCTCGAG 500

GAATTTCAGCTAGCATTTTGGTACCAATCTCCTTCAAACAAAATCTTTAAATGAGGGGTTCTTAAACTTGTTGTATACTTTAAAGAATATACTGATGCTT 600

ATCCTATGATTGCATGAATTGATCTTAATATTTTAAAAAATTTATATAAAAATTCAACAACAGTGCAAACCCAACGCAACAAAATTAATGAAAAAATAGT 700

GTGAAATCCCATGCAAAACTTGTTAAAGCTCGATATTGGGTAAATAACTTAATAGGCCTGTGAGAGAATTAGTTCAACTAAGAATGAAGGGTAGTTCTAA 800

ATTACTATTATCTCAGCAGGCAAAACATCATTTTTTTTTAGGGCTTTTGCGCGAACACGGTCAGGTGCGTATTGGACGAGTAATTTTCGTCGCCGGTTAT 900

CCTTCATCATTCTCCAATCTGTCCAATTGTTTCTGACTGGTTGATTCAATTTTACTGCATTTGTCATCTAAGAAATTTTGTTTTGTTTACGTAACTGATA 1000

TATAAAACGAAAAATATAAAAACATACTCCATTTCGAAGTAGTTGGGCGGTGTTTTGCAATAAAAAACTTGAAAAATTTAACAATTTGCTCATGGTAGTC 1100

GTGATGGCCATTTTGTTCTGAAGAGTAAACAAAGAGATGTCAAATCGACAAATCAGACTTGCAAGAAAACATTTTCAAACAACGTTAAACTTTATAGTTG 1200

TTAAGATAAAAATTTTAAAATCAAAAATTCATTTGGGAACAATGTGTTTTTTTTTTTTACCTTGAAAACAACCTTGTGCAAAGCCCCTAGAGAGGGGGAG 1300

AAATATCCGAGGGAGAAAAAAATTTAAATAACCTTGAAAGAAGCATCAGTAAATAGATTGTGGTTGTTTGATAAAATCAAAAATTAATAATCTACATTTT 1400

TCTAACATTAATCAATTGAAATGTTATAAAATTAAACAATGTTTAACGCAATGCAACTCTGTTCCCCTCCCCTGTAGAGACTTTACTCCGTAGGGGCTAG 1500

CAACTTCCAGCGTCACTCGTTCTCGTGTCTTTCAAGGTTCGTTCTCTTCTCTTCCAACGGAACATCAGGTAAGCACACGTTTTGACGCGTCTCCCAAATT 1600

TATCCTCGCAAATCGAAGTATTCTTTGTCAATCCATTAGTAACAATGTCTTCAAAGCATAGAAATACTCATTCTTCGTTTAATTAAGCTATTTTAGGATA 1700

ATAACGATGAGAAGGTGATGGGTTTTTTTGTAGAGAAAAGTTTCCAGTGCAACTGTCAAGTGGGCCGGTTTCAATCGACATGTGGGGCTTGGTCGCCATT 1800

GCGGGGCGATTTTCGTTTTCGTACTTTTCAGTTCTAATCTAGGGACAAGTTAACATTCATTACATTGAATTAATAGTTTCATGGTTTCATTCTAGACATT 1900

TTGTAGGATTTCAGTATTTCTTCATGCTGTGGCGCCTCCACTTATTTACCGGGGTGTAGTCCCAATTGGGCAATGGCTAAATCTTTCATACATTTTCACT 2000

**10F**

AATTCTTTTGAATAATTTGCCCTTGAAAATCATTTAACATTAATCTTTTTAGTTCAAGGTCGTGGGGGCGAATTTCAGTTGGGGTTAATAAACTGGTGGT 2100

TACTGGGTGTGGGGTTAGTTGCCCTCAAGTGGTTTTACTTTACTGAGGTGTGGTCATTCGATATAAATAGGGTGAATTTTGTAGGGATCTTTCATTCTCT 2200

TTTACCTTGTAGGTCGGTTGAGTTCAGCTCGTCTTCAAGAAATTTAAAGTTCTTTCTTTGTTGCAGCTGTTTCAGTTTGTTCAACCGATCCTAAACTCCA 2300

ATCACAATGCCCGGGTATGGGCAACAAATGCTGCAGCAAGCGACAGGATCAGGAACTGGCACTGGCCTATCCCACTGGGGGCTACAAGAAATCCGACTAC 2400

ACCTTTGAACAAAAACTCATCTCAGAAGAGGATCTGGTGAGCAAGGGCGAGGAGGATAACATGGCCATCATCAAGGAGTTCATGCGCTTCAAGGTGCACA 2500

**mid_R**

TGGAGGGCTCCGTGAACGGCCACGAGTTCGAGATCGAGGGCGAGGGCGAGGGCCGCCCCTACGAGGGCACCCAGACCGCCAAGCTGAAGGTGACCAAGGG 2600

TGGCCCCCTGCCCTTCGCCTGGGACATCCTGTCCCCTCAGTTCATGTACGGCTCCAAGGCCTACGTGAAGCACCCCGCCGACATCCCCGACTACTTGAAG 2700

CTGTCCTTCCCCGAGGGCTTCAAGTGGGAGCGCGTGATGAACTTCGAGGACGGCGGCGTGGTGACCGTGACCCAGGACTCCTCCCTGCAGGACGGCGAGT 2800

TCATCTACAAGGTGAAGCTGCGCGGCACCAACTTCCCCTCCGACGGCCCCGTAATGCAGAAGAAGACCATGGGCTGGGAGGCCTCCTCCGAGCGGATGTA 2900

CCCCGAGGACGGCGCCCTGAAGGGCGAGATCAAGCAGAGGCTGAAGCTGAAGGACGGCGGCCACTACGACGCTGAGGTCAAGACCACCTACAAGGCCAAG 3000

AAGCCCGTGCAGCTGCCCGGCGCCTACAACGTCAACATCAAGTTGGACATCACCTCCCACAACGAGGACTACACCATCGTGGAACAGTACGAACGCGCCG 3100

AGGGCCGCCACTCCACCGGCGGCATGGACGAGCTGTACAAGTACCCATACGATGTTCCAGATTACGCTGAGGGCAGGGGAAGTCTTCTAACATGCGGGGA 3200

CGTGGAGGAAAATCCCGGGCCCCATCACCATCACCATCACCCCCCTAAAGTAAGTGGGAAAGCAGCGAAGAAAGCTGGGAAAGCTCAGAAGAATATCACT 3300

AAAGGCGATAAAAAGAAGAAGCGTAGAAGGAAGGAGAGCTACGCAATTTACATCTACAAAGTGCTGAAGCAAGTCCATCCCGACACTGGTATTTCCTCGA 3400

AAGCCATGACGATCATGAACAGCTTCGTCAACGATATTTTCGAGCGCATTGCTGGAGAATCCTCTCGTCTTGCTCACTACAACAAGCGTTCTACCATCAC 3500

GAGTCGAGAAATCCAAACAGCTGTCCGTCTTCTTTTGCCCGGTGAATTAGCGAAACACGCCGTCTCTGAAGGCACCAAAGCTGTCACCAAGTATACCAGC 3600

ACAAAGGATCCACCGGTCGCCACCATGGTGAGCAAGGGCGAGGAGCTGTTCACCGGGGTGGTGCCCATCCTGGTCGAGCTGGACGGCGACGTAAACGGCC 3700

ACAAGTTCAGCGTGTCCGGCGAGGGCGAGGGCGATGCCACCTACGGCAAGCTGACCCTGAAGTTCATCTGCACCACCGGCAAGCTGCCCGTGCCCTGGCC 3800

CACCCTCGTGACCACCCTGACCTACGGCGTGCAGTGCTTCAGCCGCTACCCCGACCACATGAAGCAGCACGACTTCTTCAAGTCCGCCATGCCCGAAGGC 3900

TACGTCCAGGAGCGCACCATCTTCTTCAAGGACGACGGCAACTACAAGACCCGCGCCGAGGTGAAGTTCGAGGGCGACACCCTGGTGAACCGCATCGAGC 4000

TGAAGGGCATCGACTTCAAGGAGGACGGCAACATCCTGGGGCACAAGCTGGAGTACAACTACAACAGCCACAACGTCTATATCATGGCCGACAAGCAGAA 4100

GAACGGCATCAAGGTGAACTTCAAGATCCGCCACAACATCGAGGACGGCAGCGTGCAGCTCGCCGACCACTACCAGCAGAACACCCCCATCGGCGACGGC 4200

CCCGTGCTGCTGCCCGACAACCACTACCTGAGCACCCAGTCCGCCCTGAGCAAAGACCCCAACGAGAAGCGCGATCACATGGTCCTGCTGGAGTTCGTGA 4300

CCGCCGCCGGGATCACTCTCGGCATGGACGAGCTGTACAAGGACTACAAAGACGATGACGACAAGTAGATGGAGGCTACTATTCCATCCAACCGACAAGA 4400

GTGTCATGTACCGGGTCCGAAGCATCTACAGTTTGAAATTCTTCATTTAGTTTCCGTAAAAAAATTCTTCAATCGGCATAGAAAAGAATTCAGGAGAAAA 4500

GCAAGCGTTTGGTTGAAGCAACTTGTTTTCAACTTTGTTATGTAGGACTAGACTATCTGTCTACAGTATGTCTTTGCTTGTATCCTGTTGAATTTGTGTC 4600

GTATTTTCTTCCTTTAACACGAAATTTTAGCGCGTTTCCTACTATTGGAGTGGGTAATCCTGGAGAAGAATAATGATATCTTGGTTTGATTAATTGAAAA 4700

ACAGCGCCCTCGTGTACTTTCTGAGTGCATTTTACTTTTATTAGTCATCTTGTACATTATTGAGGTGGCTTTATTGGTAAATTGTTAACTTTCCACTTGG 4800

TTTATTCGAATCGTTTTTACTTACTCCCTGTATGTGTAAGGGCTTTGGTATATGCACCTCAATCAAGCTCCAATACAAGATAATTTGGACCCCGGTACCC 4900

AGCTTTTGTTCCCTTGTGACGATGTGATGTCGGACGACGGTGGCGTCAGTTCCGGCGAGAATTCTCGGTCGGCTCCGCCCACGCCGACGACAACAACACC 5000

TTCGGACGACGATCAGGCTCGACTGCGACTCAAGCGCAAACTCCAGCGCAACCGCACTTCCTTCACCAACGAACAGATCGAGAGCCTCGTTAATTGCGCG 5100

CTTGGCGTAATCATGGTCATAGCTGTTTCCTGTGTGAAATTGTTATCCGCTCACAATTCCACGAACCGTAAAAAGGCCGCGTTGCTGGCGTTTTTCCATA 5200

GGCTCCGCCCCCCTGACGAGCATCACAAAAATCGACGCTCAAGTCAGAGGTGGCGAAACCCGACAGGACTATAAAGATACCAGGCGTTTCCCCCTGGAAG 5300

CTCCCTCGTGCGCTCTCCTGTTCCGACCCTGCCGCTTACCGGATACCTGTCCGCCTTTCTCCCTTCGGGAAGCGTGGCGCTTTCTCATAGCTCACGCTGT 5400

AGGTATCTCAGTTCGGTGTAGGTCGTTCGCTCCAAGCTGGGCTGTGTGCACGAACCCCCCGTTCAGCCCGACCGCTGCGCCTTATCCGGTAACTATCGTC 5500

TTGAGTCCAACCCGGTAAGACACGACTTATCGCCACTGGCAGCAGCCACTGGTAACAGGATTAGCAGAGCGAGGTATGTAGGCGGTGCTACAGAGTTCTT 5600

GAAGTGGTGGCCTAACTACGGCTACACTAGAAGAACAGTATTTGGTATCTGCGCTCTGCTGAAGCCAGTTACCTTCGGAAAAAGAGTTGGTAGCTCTTGA 5700

TCCGGCAAACAAACCACCGCTGGTAGCGGTGGTTTTTTTGTTTGCAAGCAGCAGATTACGCGCAGAAAAAAAGGATCTCAAGAAGATCCTTTGATCTTTT 5800

CTACGGGGTCTGACGCTCAGTGGAACGAAAACTCACGTTAAGGGATTTTGGTCATGAGATTATCAAAAAGGATCTTCACCTAGATCCTTTTAAATTAAAA 5900

ATGAAGTTTTAAATCAATCTAAAGTATATATGAGTAAACTTGGTCTGACAGTTACCAATGCTTAATCAGTGAGGCACCTATCTCAGCGATCTGTCTATTT 6000

CGTTCATCCATAGTTGCCTGACTCCCCGTCGTGTAGATAACTACGATACGGGAGGGCTTACCATCTGGCCCCAGTGCTGCAATGATACCGCGAGACCCAC 6100

GCTCACCGGCTCCAGATTTATCAGCAATAAACCAGCCAGCCGGAAGGGCCGAGCGCAGAAGTGGTCCTGCAACTTTATCCGCCTCCATCCAGTCTATTAA 6200

TTGTTGCCGGGAAGCTAGAGTAAGTAGTTCGCCAGTTAATAGTTTGCGCAACGTTGTTGCCATTGCTACAGGCATCGTGGTGTCACGCTCGTCGTTTGGT 6300

ATGGCTTCATTCAGCTCCGGTTCCCAACGATCAAGGCGAGTTACATGATCCCCCATGTTGTGCAAAAAAGCGGTTAGCTCCTTCGGTCCTCCGATCGTTG 6400

TCAGAAGTAAGTTGGCCGCAGTGTTATCACTCATGGTTATGGCAGCACTGCATAATTCTCTTACTGTCATGCCATCCGTAAGATGCTTTTCTGTGACTGG 6500

TGAGTACTCAACCAAGTCATTCTGAGAATAGTGTATGCGGCGACCGAGTTGCTCTTGCCCGGCGTCAATACGGGATAATACCGCGCCACATAGCAGAACT 6600

TTAAAAGTGCTCATCATTGGAAAACGTTCTTCGGGGCGAAAACTCTCAAGGATCTTACCGCTGTTGAGATCCAGTTCGATGTAACCCACTCGTGCACCCA 6700

ACTGATCTTCAGCATCTTTTACTTTCACCAGCGTTTCTGGGTGAGCAAAAACAGGAAGGCAAAATGCCGCAAAAAAGGGAATAAGGGCGACACGGAAATG 6800

TTGAATACTCATACTCTTCCTTTTTCAATATTATTGAAGCATTTATCAGGGTTATTGTCTCATGAGCGGATACATATTTGAATGTATTTAGAAAAATAAA 6900

CAAATAGGGGTTCCGCGCACATTTCCCCGAAAAGTGCCACCTAAATTGTAAGCGTTAATATTTTGTTAAAATTCGCGTTAAATTTTTGTTAAATCAGCTC 7000

ATTTTTTAACCAATAGGCCGAAATCGGCAAAATCCCTTATAAATCAAAAGAATAGACCGAGATAGGGTTGAGTGTTGTTCCAGTTTGGAACAAGAGTCCA 7100

CTATTAAAGAACGTGGACTCCAACGTCAAAGGGCGAAAAACCGTCTATCAGGGCGATGGCCCACTACGTGAACCATCACCCTAATCAAGTTTTTTGGGGT 7200

CGAGGTGCCGTAAAGCACTAAATCGGAACCCTAAAGGGAGCCCCCGATTTAGAGCTTGACGGGGAAAGCCGGCGAACGTGGCGAGAAAGGAAGGGAAGAA 7300

**1F**

AGCGAAAGGAGCGGGCGCTAGGGCGCTGGCAAGTGTAGCGGTCACGCTGCGCGTAACCACCACACCCGCCGCGCTTAATGCGCCGCTACAGGGCGCGTCC 7400

CATTCGCCATTCAGGCTGCGCAACACAGGTTTGGTTCAGTAACCGACGAGCAAAATGGCGTCGTGAGGAGAAATTACGGAATCAACGGCGTGGACCAGAA 7500

CAACAACAATCGTCGTCGCAACAGCCGGATCAAGTCAATCCCCAGCAGCAGGATGGGTGAGGTGGAGTACGCGCCCGGGGAGCCCAAGGGCACGCCCTGG 7600

TCACAATGCCCGGGTCACCCGCACCGCGGCTTCGAGGTCGACATAACTTCGTATAATGTATGCTATACGAAGTTATA -3’

**S2 Fig. Full sequence of donor DNA.** Arrows indicate the positions of the primers used for genomic polymerase chain reaction.
