# Supplementary material for: CRISPR/Cas-mediated knock-in via non-homologous end-joining in the crustacean Daphnia magna
Source: PLoS One. 2017 Oct 18;12(10):e0186112. doi: 10.1371/journal.pone.0186112 (PMC5646780; doi:10.1371/journal.pone.0186112)
Supplement: S3 Fig — WT shows the original Dma-ey targeting gRNA-2 sequence. TG 1st and TG 2nd show the first and second target sites of the transgene region in the same orientation as the endogenous eyeless gene. No mutation was found in either of the target sites. (DOCX) [file pone.0186112.s003.docx]

WT 5’-TGGCGTCGTGAGGAGAAATTA-3’

TG 1st 5’-TGGCGTCGTGAGGAGAAATTA-3’

TG 2nd 5’-TGGCGTCGTGAGGAGAAATTA-3’

**S3 Fig. Comparison of the Cas9 target sites in the transgene region with the original sequence.** WT shows the original *Dma-ey* targeting gRNA-2 sequence. TG 1st and TG 2nd show the first and second target sites of the transgene region in the same orientation as the endogenous *eyeless* gene. No mutation was found in either of the target sites.
